# Factors associated with prolonged length of stay in the psychiatric emergency service

**DOI:** 10.1371/journal.pone.0202569

**Published:** 2018-08-20

**Authors:** Chun-Chi Hsu, Hung-Yu Chan

**Affiliations:** 1 Department of General Psychiatry, Taoyuan Psychiatric Center, Ministry of Health and Welfare, Taoyuan, Taiwan; 2 Department of Psychiatry, National Taiwan University Hospital and College of Medicine, National Taiwan University, Taipei, Taiwan; Department of Psychiatry and Neuropsychology, Maastricht University Medical Center, NETHERLANDS

## Abstract

**Objective:**

Dedicated regional psychiatric emergency services (PES) were proposed as a better care model for psychiatric emergencies and a possible solution to boarding of psychiatric patients in the emergency department. However, there are limited data on factors associated with prolonged length of stay (LOS) in the PES. The objective of this study was finding factors associated with prolonged LOS in the PES and moving towards a solution to this problem.

**Methods:**

The study sample comprised 200 PES visits randomly chosen from January 2011 to December 2015 in a psychiatric hospital in Taiwan. Relevant data were collected comprehensively through the health information system and by reviewing medical records. The primary outcome was LOS longer than 24 hours while LOS longer than 48 hours was used as the secondary outcome.

**Results:**

Mean LOS was 17.6±23.2 hours, with 53 (26.5%) visits lasting more than 24 hours and 15 (7.5%) visits lasting more than 48 hours. After adjusting for related confounders, LOS longer than 24 hours was associated with use of restraints in the PES (adjusted odds ratio (aOR) = 3.13, 95% CI = 1.59–6.15) and history of illicit substance use (aOR = 2.46, 95% CI = 1.11–5.44). LOS longer than 48 hours was associated with use of restraints in the PES (aOR = 4.11, 95% CI = 1.2–14.14), history of illicit substance use (aOR = 6.16, 95% CI = 1.37–27.62) and first time visit to the hospital (aOR = 8.54, 95% CI = 2.03–35.96). Neither outcome was associated with transfer to an inpatient unit.

**Conclusion:**

Prolonged LOS was common in the study sample. Discharged patients had an equally high rate of prolonged LOS as admitted patients. Therefore measures should be taken to facilitate timely discharge. Use of restraints and history of illicit substance use were common among patients with prolonged LOS.

## Introduction

In recent years, boarding of psychiatric patients in the emergency department (ED) has been recognized as a significant problem in the US [1–3]. Boarding is often defined as the practice of holding admitted patients in the ED until an inpatient bed becomes available [1,4–7]. However, there has not been a standard definition of boarding in the case of psychiatric patients [1]. The American College of Emergency Physician classified psychiatric boarding as remaining in the ED for four or more additional hours after the decision to admit is made [2]. Boarded patients often receive little active treatment in the ED due to limitations of the setting, and the often crowded and noisy environment may actually worsen their condition [8]. Dedicated regional psychiatric emergency services (PES) have been proposed as a better model for delivering care for psychiatric emergencies and a possible solution for boarding of psychiatric patients in the ED [9–11].

A PES is a specialized ED that provides 24 hour emergency psychiatric care. Emergency psychiatry is typically practiced in two models [12,13]. The “Triage Model” comprises rapid evaluation, containment and referral. It is also the model adopted by most general ED. The psychiatric consultant often only has the choice between discharge and inpatient admission with little option for treatment because of the ED setting. Whereas a standalone PES follows the “Treatment Model,” which has the capacity for treating the patient onsite [14]. Studies have found that with prompt intervention, the majority of psychiatric emergencies can be resolved in less than 24 hours, with no need for inpatient admission [9,10,15]. This is beneficial to the patient because of the shorter hospital stay, and inpatient bed availability is preserved.

The “Alameda Model” described by Zeller et al. demonstrated that a dedicated regional PES decreased boarding time for psychiatric patients in area general ED. ED boarding time for psychiatric patients in Alameda County, California was reduced by 80% versus comparable state ED average once a regional PES was established [10]. Psychiatric patients can be transferred from area general ED for evaluation and treatment after medically cleared. The PES can also accept ambulance and police deliveries directly, as well as self-referrals. Little-Upah et al. reported a similar program in Phoenix, Arizona [9]. The Comprehensive Psychiatric Emergency Programs in New York are also examples of dedicated regional PES [16]. There are several hospitals in Taiwan with similar PES programs, including Taoyuan Psychiatric Center (the study hospital), Taipei City Hospital Songde Branch and Kai-Syuan Psychiatric Hospital.

However, PES is by no means a perfect solution and still faces many of the same problems a general ED does, including crowding. In recent years, ED crowding has become an international public health crisis [17,18]. Crowding exhausts the already scarce ED resources and causes treatment delay, ambulance diversion, longer waiting times for patients and lower patient satisfaction [18,19]. Poorer patient outcomes, including more adverse events and longer hospital stays, have also been widely reported to be associated with a crowded ED [5,20,21]. Multiple contributors to ED crowding have been proposed. Studies have shown that factors such as repeated or inappropriate ED visits were not as significant as previously thought [22,23]. However, prolonged ED length of stay (LOS) has been one of the most widely accepted associating factors [24,25] and ED LOS is now one of the primary measures of ED quality of care [26–28].

ED crowding is also a major problem of the healthcare system in Taiwan. Rates of prolonged ED LOS have risen to unprecedented levels in many hospitals and have received substantial attention from local popular press. It is not unheard of that a patient stays in the ED for days on end, especially in medical centers. Patients generally stay longer in the ED in Taiwan than in the US [29–31]. In the US, the Centers for Medicare and Medicaid Services states "only in rare and exceptional circumstances would it be reasonable and necessary for outpatient observation services to span more than 48 hours" [32]. Hospitals in the US face increasing costs without additional revenue if a patient stays in the ED beyond 48 hours. In contrast, the National Health Insurance in Taiwan reimburses hospitals for each additional day a patient stays in the ED.

To the best of our knowledge, only one study analyzed factors associated with prolonged LOS in the PES [33]. It included only limited factors and was a case-control study; therefore did not have data on the rate of prolonged LOS. In that study, prolonged LOS was associated with suicidal ideation, disposition to an inpatient unit, homicidal ideation, lack of insurance, homelessness, male gender, past history of psychiatric hospitalization, diagnosis of substance abuse, significant psychiatric co-morbidity (represented by three or more Axis I diagnoses), and diagnosis of a psychotic disorder. Another study was also conducted in the PES but used boarding as the primary outcome [34]. The study concluded that boarding was associated with referral by a party other than the patient, arrival on or just after the weekend, arrival in restraints, physical restraint or seclusion in the PES, referral for involuntary hospitalization, primary anxiety disorders or personality disorders, and tobacco use. Although boarding has been identified as an important contributing factor of prolonged LOS in general ED [35], factors associated with boarding may not necessarily be the same as those associated with prolonged LOS in the PES. Currently data on prolonged LOS and crowding in the PES are sparse as few studies were conducted in the PES. In addition, past studies shed little light on this matter for they either were limited by study design and selection of risk factors or had a different study focus. The present study aimed for a more exhaustive analysis of factors associated with prolonged LOS in the PES and would further the knowledge of the extent of crowding in the PES and possible reasons behind it.

## Methods

### Study setting and population

The study site is a teaching psychiatric hospital in northern Taiwan with 282 acute care beds and 380 chronic care beds. The PES is an individual ten-bed locked psychiatric emergency facility attached to the main hospital building. It provides around-the-clock, year-round emergency psychiatric care to medically stable patients of all ages and diagnoses. Those who are medically unstable are transferred to nearby general hospitals. The main treatment goals of the PES are to provide effective assessment, rapid stabilization and appropriate placement of patients. It is equipped with a staff composed of an attending psychiatrist, a resident psychiatrist and nurses at all times. Social workers are also available during standard working hours to provide necessary assistance. The annual census is approximately 2600 visits. This study was reviewed and approved by the Ethics Committee of Taoyuan Psychiatric Center (IRB number: B20170111), and the requirement for informed consent was waivered by the Ethics Committee.

### Study design

#### Sample selection and sample size calculation

Two hundred PES visits were randomly chosen from a period of five years (January 2011, through December 2015) and comprehensively reviewed. Data of all PES visits of this time frame were abstracted from the health information system first, and then a random sample of 200 visits was generated using the sampling analysis tool in Microsoft Excel. Due to the exploratory nature of this study, we made no a priori assumption regarding the probable risk factors of prolonged LOS in the PES. The sample size determination of this study was based on the work of Peduzzi et al [36]. Their findings recommended that logistic regression should be used with a minimum of 10 events per variable. Monthly average LOS and rate of prolonged LOS in the PES have been monitored at the study hospital for years as a measure of quality assurance. Rates of LOS over 24 hours usually range from 20 to 25%. We made a conservative estimate that the proportion of visits with LOS over 24 hours in the study sample would be around 20%, and there would be four independent variables in the final regression model. Therefore we determined that at least 200 PES visits were needed for this study (n = 10x4/0.2 = 200).

#### Explanatory variables

For each visit, patient age and gender, ICD-9-CM billing codes, date and time of arrival, LOS and disposition were abstracted electronically from the health information system. Further clinical details of each visit and history of the patient were obtained by reviewing paper records. Clinical details included mode of arrival, use of restraints and referral for involuntary hospitalization. History of the patient included marital status, living arrangement, education level, employment status, age of onset, previous visit and inpatient admission to the hospital, self-injury, violence and substance misuse (i.e. cigarette, alcohol, prescription drug and illicit substance). All the information was originally collected as part of routine care and fully anonymized prior to statistical analysis. A trained research assistant under the supervision of a board-certified psychiatrist collected and categorized the data. Regular meetings were held to discuss progress, and the psychiatrist was immediately consulted when uncertainty arose.

The primary diagnosis was defined as the first ICD-9-CM billing code. Psychiatric comorbidity was defined as presence of more than one ICD-9-CM billing code for psychiatric disorder (290.xx to 319.xx). Physical comorbidity was confirmed when an ICD-9-CM billing code for non-psychiatric disorder was present or when it was recorded in the chart.

In Taiwan, temporary psychiatric hold can only be initiated by a board-certified psychiatrist at a designated psychiatric facility and cannot last longer than two calendar days. Request for involuntary hospitalization must be made before temporary psychiatric hold ends, or the patient has to be released. A committee within the Ministry of Health and Welfare reviews and authorizes all requests for involuntary hospitalization. Records of these requests are kept in the corresponding charts.

Insurance status is not reported because all patients were covered by the National Health Insurance, which is government-mandated in Taiwan. Ethnicity is also not reported. Since the most common ethnicity in Taiwan is Han-Chinese which makes up over 95% of the population, ethnicity is not routinely recorded during clinical care. The American College of Emergency Physician definition for psychiatric boarding was used in this study.

#### Primary outcome and secondary outcome

LOS was defined as the time from arrival to leaving the PES, regardless of disposition, for patients who were treated in the PES. Even though some countries, such as the UK introduced a four-hour target for LOS in the ED, this study used LOS longer than 24 hours as the primary outcome to be consistent with prior literature [4,33,37,38]. The target for LOS in the ED set by the National Health Insurance in Taiwan is 48 hours; therefore LOS longer than 48 hours was used as the secondary outcome.

### Statistical analysis

Continuous data are presented as means and standard deviations, while categorical data are presented as observed numbers and percentages. Demographic and clinical characteristics of PES visits were analyzed using Student's t-test, chi-squared test and logistic regression. Covariates were included in the regression model if they had a p-value less than 0.05 in univariate comparison or were deemed to be of clinical significance, including gender, age, living arrangement, employment status, primary diagnosis, physical comorbidity, month of visit, weekend or holiday visit, night shift visit, mode of arrival, arrival in restraints, use of restraints in the PES, transfer to an inpatient unit, referral for involuntary hospitalization, first time visit to the hospital, history of self-injury, history of violence, history of alcohol misuse and history of illicit substance use. Forward logistic regression was used for selection of variables in the final regression model. The appropriateness of the logistic regression model was tested using the Hosmer-Lemeshow test. The level of significance was set at 5%. All analyses were conducted using Statistical Analytic Software version 9.3 (SAS Institute Inc., Cary, North Carolina, USA).

## Results

There were in total 13373 PES visits during the study period. Of those, 200 visits were randomly chosen, and none were excluded. No two visits were made by the same patient. The demographic and clinical characteristics are shown in Table 1. The visits were evenly distributed across the five year span with the yearly breakdown ranging from 17.5% to 24.5%. The mean age of these patients was 41.5 ± 15.7 years, with slight more than half (53%, 106/200) being of the male gender. The mean LOS was 17.6 ± 23.2 hours, with 53 (26.5%, 53/200) visits lasting more than 24 hours and fifteen (7.5%, 15/200) visits lasting more than 48 hours. Eighty-six (43%, 86/200) patients arrived by ambulance. The most common diagnosis was mood disorder (49.5%, 99/200), followed by schizophrenia (34%, 68/200). Twenty-five (12.5%, 25/200) patients arrived in restraints, and 59 (29.5%, 59/200) patients experienced physical restraint in the PES. Involuntary hospitalization was requested for 3 (1.5%, 3/200) patients.

**Table 1:** Demographic and clinical characteristics of all visits.

	N = 200	%
**Gender**		
Male	106	53%
Female	94	47%
**Age**		
Years, mean ± SD	41.5 ± 14.0	
**Marital Status**		
Single	100	50%
Married	64	32%
Divorced	25	12.5%
Widowed	11	5.5%
**Living Arrangement**		
Lived alone	27	13.5%
Lived with family	166	83%
Other	7	3.5%
**Education Level**		
Elementary school	27	14%
Middle school	45	22.5%
High school	85	42.5%
College	37	18.5%
Graduate school	6	3%
**Employment**		
No	142	71%
Yes	58	29%
**Age of Onset**		
Years, mean ± SD	31.7 ± 15.4	
**Age of First Time Visit to the Hospital**		
Years, mean ± SD	35.5 ± 15.7	
**Previous Inpatient Admissions to the Hospital**		
Frequency, mean ± SD	2.4 ± 4.1	
**Primary Diagnosis (ICD-9-CM Code)**		
Schizophrenia (295.xx)	68	34%
Mood disorder (296.xx)	99	49.5%
Others	33	16.5%
**Psychiatric Comorbidity**		
No	96	48%
Yes	104	52%
**Physical Comorbidity**		
No	144	72%
Yes	56	28%
**Family History of Psychiatric Disorders**		
No	154	77%
Yes	46	23%
**Date of Visit** **Year**		
2011	49	24.5%
2012	37	18.5%
2013	35	17.5%
2014	39	19.5%
2015	40	20%
**Month**		
Jan/Feb/Mar	49	24.5%
Apr/May/June	50	25%
July/Aug/Sept	49	24.5%
Oct/Nov/Dec	52	26%
**Visited on a Weekend or Holiday**		
No	144	72%
Yes	56	28%
**Visited during Night Shift (Midnight to 8 AM)**		
No	169	84.5%
Yes	31	15.5%
**Arrival by Ambulance**		
No	114	57%
Yes	86	43%
**Arrived in Restraints**		
No	175	87.5%
Yes	25	12.5%
**Experienced Restraints in the PES**		
No	141	70.5%
Yes	59	29.5%
**Transferred to an Inpatient Unit**		
No	104	52%
Yes	96	48%
**Referred for Involuntary Hospitalization**		
No	197	98.5%
Yes	3	1.5%
**First Time Visit**		
No	154	77%
Yes	46	23%
**History of Self-Injury**		
No	116	58%
Yes	84	42%
**History of Violence**		
No	73	36.5%
Yes	127	63.5%
**History of Alcohol Misuse**		
No	132	67%
Yes	68	34%
**History of Cigarette Smoking**		
No	127	63.5%
Yes	73	36.5%
**History of Illicit Substance Use**		
No	163	81.5%
Yes	37	18.5%
**History of Prescription Drug Misuse**		
No	185	92.5%
Yes	15	7.5%
**LOS** **≧24 Hours**		
No	147	73.5%
Yes	53	26.5%
**≧48 Hours**		
No	185	92.5%
Yes	15	7.5%
**LOS**		
hh:mm, mean ± SD	17:34 ± 23:13	

Abbreviations: PES = Psychiatric Emergency Service, LOS = Length of Stay, hh:mm = Hour:Minute.

Of all the patients, 79 (39.5%, 79/200) were discharged within 24 hours, and 95 (47.5%, 95/200) were discharged within 48 hours. Ultimately 104 (52%, 104/200) patients were discharged from the PES, and 96 (48%, 96/200) were transferred to an inpatient unit. The mean LOS was 16.37 ±24.13 hours for patients discharged from the PES and 18.9 ± 22.2 hours for patients transferred to an inpatient unit. Among the patients discharged from the PES, 25 (24.0%, 25/104) stayed in the PES for more than 24 hours, and nine (8.7%, 9/104) for more than 48 hours. Among the patients transferred to an inpatient unit, 28 (29.2%, 28/96) stayed in the PES for more than 24 hours and six (6.3%, 6/96) for more than 48 hours.

Table 2 shows the results of univariate comparisons when prolonged LOS was defined as LOS longer than 24 hours. It was found that prolonged LOS was associated with arrival by ambulance (p = 0.01), arrival in restraints (p = 0.03), use of restraints in the PES (p<0.01), referral for involuntary hospitalization (p<0.01) and history of illicit substance use (p = 0.03). Logistic regression analysis revealed that LOS longer than 24 hours was associated with use of restraints in the PES (adjusted odds ratio (aOR) = 3.13, 95% CI = 1.59–6.15, p<0.01) and history of illicit substance use (aOR = 2.46, 95% CI = 1.11–5.44, p = 0.03). The model was appropriately fitted (Hosmer-Lemeshow statistic = 0.07, degree of freedom = 2, p = 0.97; Nagelkerke R^2^ = 0.15).

**Table 2:** Univariate comparisons between visits with LOS ≧ 24hours and LOS < 24 hours.

	LOS ≧24hrs N = 53	%	LOS <24hrs N = 147	%	p-value^a^
**Gender**					0.98
Male	28	52.8%	78	53.1%	
Female	25	47.2%	69	46.9%	
**Age**					0.16
Years, mean ± SD	39.2 ± 14.0		42.3 ± 13.9		
**Marital Status**					0.36
Single	32	60.4%	68	46.3%	
Married	14	26.4%	50	34%	
Divorced	5	9.4%	20	13.6%	
Widowed	2	3.8%	9	6.1%	
**Living Arrangement**					0.91
Lived alone	8	15.1%	19	12.9%	
Lived with family	43	81.1%	123	83.7%	
Others	2	3.8%	5	3.4%	
**Education Level**					0.78
Elementary school	6	11.3%	21	14.3%	
Middle school	15	28.3%	30	20.4%	
High school	21	39.6%	64	43.5%	
College	10	18.9%	27	18.4%	
Graduate school	1	1.9%	5	3.4%	
**Employment**					0.12
No	42	79.2%	100	68%	
Yes	11	20.8%	47	32%	
**Age of Onset**					0.86
Years, mean ± SD	31.3 ± 15.6		31.8 ± 15.4		
**Age of First Time Visit to the Hospital**					0.41
Years, mean ± SD	34.0 ± 15.5		36.1 ± 15.7		
**Previous Inpatient Admissions to the Hospital**					0.57
Frequency, mean ± SD	2.1 ± 2.9		2.5 ± 4.4		
**Primary Diagnosis (ICD-9-CM Code)**					0.27
Schizophrenia (295.xx)	16	30.2%	52	35.4%	
Mood disorder (296.xx)	31	58.5%	68	46.3%	
Other	6	11.3%	27	18.4%	
**Psychiatric Comorbidity**					0.89
No	25	47.2%	71	48.3%	
Yes	28	52.8%	76	51.7%	
**Physical Comorbidity**					0.68
No	37	69.8%	107	72.8%	
Yes	16	30.2%	40	27.2%	
**Family History of Psychiatric Disorders**					0.76
No	40	75.5%	114	77.6%	
Yes	13	24.5%	33	22.4%	
**Date of Visit** **Year**					0.06
2011	10	18.9%	39	26.5%	
2012	6	11.3%	31	21.1%	
2013	7	13.2%	28	19%	
2014	14	26.4%	25	17%	
2015	16	30.2%	24	16.3%	
**Month**					0.55
Jan/Feb/Mar	12	22.6%	37	25.2%	
Apr/May/June	10	18.9%	40	27.2%	
July/Aug/Sept	15	28.3%	34	23.1%	
Oct/Nov/Dec	16	30.2%	36	24.5%	
**Visited on a Weekend or Holiday**					0.31
No	41	77.4%	103	70.1%	
Yes	12	22.6%	44	29.9%	
**Visited during Night Shift (Midnight to 8 AM)**					0.22
No	42	79.2%	127	86.4%	
Yes	11	20.8%	20	13.6%	
**Arrival by Ambulance**					0.01*
No	22	41.5%	92	62.6%	
Yes	31	58.5%	55	37.4%	
**Arrived in Restraints**					0.03*
No	42	79.2%	133	90.5%	
Yes	11	20.8%	14	9.5%	
**Experienced Restraints in the PES**					<0.001***
No	28	52.8%	113	76.9%	
Yes	25	47.2%	34	23.1%	
**Transferred to an Inpatient Unit**					0.41
No	25	47.2%	79	53.7%	
Yes	28	52.8%	68	46.3%	
**Referred for Involuntary Hospitalization**					<0 .01**
No	50	94.3%	147	100%	
Yes	3	5.7%	0	0%	
**First Time Visit**					0.07
No	36	67.9%	118	80.3%	
Yes	17	32.1%	29	19.7%	
**History of Self-Injury**					0.29
No	34	64.2%	82	55.8%	
Yes	19	35.8%	65	44.1%	
**History of Violence**					0.27
No	16	30.2%	57	38.8%	
Yes	37	69.8%	90	61.2%	
**History of Alcohol Misuse**					0.31
No	32	60.4%	100	68%	
Yes	21	39.6%	47	32%	
**History of Cigarette Smoking**					0.91
No	34	64.2%	93	63.3%	
Yes	19	35.8%	54	36.7%	
**History of Illicit Substance Use**					0.03*
No	38	71.7%	125	85%	
Yes	15	28.3%	22	15%	
**History of Prescription Drug Misuse**					0.07
No	52	98.1%	133	90.5%	
Yes	1	1.9%	14	9.5%	
**LOS**					
hh:mm, mean (±SD)	48:16 ± 27:04		7:08 ± 6:44		

*p-value <0 .05

**p-value <0 .01

***p-value <0 .001

Abbreviations: LOS = Length of Stay, PES = Psychiatric Emergency Service, hh:mm = Hour:Minute.

^a^Continuous data were analyzed using Student’s t test; categorical data were analyzed using chi-squared test

Table 3 shows the results of univariate comparisons when prolonged LOS was defined as LOS longer than 48 hours. It was found that prolonged LOS was associated with arrival in restraints (p = 0.01), use of restraints in the PES (p<0.01), unemployment (p = 0.05) and first time visit to the hospital (p<0.01). Logistic regression analysis revealed that LOS longer than 48 hours was associated with use of restraints in the PES (aOR = 4.11, 95% CI = 1.2–14.14, p = 0.03), first time visit to the hospital (aOR = 8.54, 95% CI = 2.03–35.96, p<0.01) and history of illicit substance use (aOR = 6.16, 95% CI = 1.37–27.62, p = 0.02). The model was appropriately fitted (Hosmer-Lemeshow statistic = 6.43, degree of freedom = 6, p = 0.38; Nagelkerke R^2^ = 0.31). Table 4 summarizes the results of the two logistic regression models, showing factors associated with LOS longer than 24 hours and 48 hours, respectively.

**Table 3:** Univariate comparisons between visits with LOS ≧ 48 hours and LOS < 48 hours.

	LOS ≧48hrs N = 15	%	LOS <48hrs N = 185	%	p-value^a^
**Gender**					0.61
Male	7	46.7%	99	53.5%	
Female	8	53.3%	86	46.5%	
**Age**					0.29
Years, mean ± SD	37.8 ± 15.4		41.8 ± 13.9		
**Marital Status**					0.47
Single	10	66.7%	90	48.6%	
Married	3	20%	61	33%	
Divorced	2	13.3%	23	12.4%	
Widowed	0	0%	11	5.9%	
**Living Arrangement**					0.56
Lived alone	3	20%	24	13%	
Lived with family	11	73.3%	155	83.8%	
Other	1	6.7%	6	3.2%	
**Education Level**					0.75
Elementary school	1	6.7%	26	14.1%	
Middle school	5	33.3%	40	21.6%	
High school	6	40%	79	42.7%	
College	3	20%	34	18.4%	
Graduate school	0	0%	6	3.2%	
**Employment**					0.05*
No	14	93.3%	128	69.2%	
Yes	1	6.7%	57	30.8%	
**Age of Onset**					0.76
Years, mean ± SD	30.5 ± 18.26		31.7 ± 15.2		
**Age of First Time Visit to the Hospital**					0.66
Years, mean ± SD	33.8 ± 17.95		35.7 ± 15.5		
**Previous Inpatient Admissions to the Hospital**					0.97
Frequency, mean ± SD	2.4 ± 3.7		2.4 ± 4.1		
**Primary Diagnosis (ICD-9-CM Code)**					0.15
Schizophrenia (295.xx)	3	20%	65	35.1%	
Mood disorder (296.xx)	11	73.3%	88	47.6%	
Other	1	6.7%	32	17.3%	
**Psychiatric Comorbidity**					0.91
No	7	46.7%	89	48.1%	
Yes	8	53.3%	96	51.9%	
**Physical Comorbidity**					0.63
No	10	66.7%	134	72.4%	
Yes	5	33.3%	51	27.6%	
**Family History of Psychiatric Disorders**					0.36
No	13	86.7%	141	76.2%	
Yes	2	13.3%	44	23.8%	
**Date of Visit** **Year**					0.09
2011	2	13.3%	47	25.4%	
2012	1	6.7%	36	19.5%	
2013	1	6.7%	34	18.4%	
2014	6	40%	33	17.8%	
2015	5	33.3%	35	18.9%	
**Month**					0.54
Jan/Feb/Mar	5	33.3%	44	23.8%	
Apr/May/June	2	13.3%	48	25.9%	
July/Aug/Sep	5	33.3%	44	23.8%	
Oct/Nov/Dec	3	20%	49	26.5%	
**Visited on a Weekend or Holiday**					0.19
No	13	86.7%	131	70.8%	
Yes	2	13.3%	54	29.2%	
**Visited during Night Shift (Midnight to 8 AM)**					0.21
No	11	73.3%	158	85.4%	
Yes	4	26.7%	27	14.6%	
**Arrival by Ambulance**					0.79
No	8	53.3%	106	57.3%	
Yes	7	46.7%	79	42.7%	
**Arrived in Restraints**					0.01*
No	10	66.7%	165	89.2%	
Yes	5	33.3%	20	10.8%	
**Experienced Restraints in the PES**					<0.001***
No	5	33.3%	136	73.5%	
Yes	10	66.7%	49	26.5%	
**Transferred to an Inpatient Unit**					0.52
No	9	60%	95	51.4%	
Yes	6	40%	90	48.6%	
**Referred for Involuntary Hospitalization**					0.09
No	14	93.3%	183	98.9%	
Yes	1	6.7%	2	1.1%	
**First Time Visit**					<0 .01**
No	7	46.7%	147	79.5%	
Yes	8	53.3%	38	20.5%	
**History of Self-Injury**					0.21
No	11	73.3%	105	56.8%	
Yes	4	26.7%	80	43.2%	
**History of Violence**					0.41
No	4	26.7%	69	37.3%	
Yes	11	73.3%	116	62.7%	
**History of Alcohol Misuse**					0.96
No	10	66.7%	122	65.9%	
Yes	5	33.3%	63	34.1%	
**History of Cigarette Smoking**					0.77
No	9	60%	118	63.8%	
Yes	6	40%	67	36.2%	
**History of Illicit Substance Use**					0.12
No	10	66.7%	153	82.7%	
Yes	5	33.3%	32	17.3%	
**History of Prescription Drug Misuse**					0.25
No	15	100%	170	91.9%	
Yes	0	0%	15	8.1%	
**LOS**					-
hh:mm, mean ± SD	83:15 ± 23:31		12:09 ± 12:25		

*p-value < 0.05

**p-value < 0.01

***p-value < 0.001

Abbreviations: LOS = Length of Stay, PES = Psychiatric Emergency Service, hh:mm = Hour:Minute.

^a^Continuous data were analyzed using Student’s t test; categorical data were analyzed using chi-squared test.

**Table 4:** Factors associated with prolonged LOS in the logistic regression models.

Variable	Adjusted Odds Ratio (95% CI)
**LOS longer than 24 hours**	
Use of restraints in the PES	3.13 (1.59–6.15)
History of illicit substance use	2.46 (1.11–5.44)
**LOS longer than 48 hours**	
Use of restraints in the PES	4.11 (1.2–14.14)
History of illicit substance use	8.54 (2.03–35.96)
First time visit to the hospital	6.16 (1.37–27.62)

Abbreviations: LOS = Length of Stay, PES = Psychiatric Emergency Service.

## Discussion

The main finding of this study was that inpatient admission was not associated with prolonged LOS in the PES in the study sample. This is in contrast to the results of past studies and may have important clinical implications. In previous studies, transfer to an inpatient unit has consistently been a significant factor associated with prolonged LOS of psychiatric patients in the general ED or the PES [4,33,37,38]. At first glance, one plausible explanation would be that boarding was less severe at the study hospital, though upon further scrutiny this is an unlikely scenario. In our experience, it is not unusual for patients to wait in the PES for an acute care bed to become available after the decision for inpatient admission has been made. Limited availability of acute care beds was believed by most staff members at the study hospital to be the most significant factor contributing to prolonged LOS and crowding in the PES because inpatient occupancy rate usually exceeds 95% all year long. Surprisingly, the findings of this study did not support such view. At the study hospital, emergency patients are generally seen by a psychiatrist within half an hour of arrival. Even when additional laboratory tests are ordered, the decision to admit can be made in less than four hours. However, the average LOS in the PES of patients transferred to an inpatient unit in this study was 18.9 hours, and 29.2% of these patients stayed in the PES longer than 24 hours. These results indicate that boarding was not insignificant at the study hospital.

A more likely explanation is the unusually prolonged LOS of discharged patients obscured the effect of inpatient admission. One study reported that boarding not only increased LOS of admitted patients in the general ED, but also LOS of discharged patients [39]. Delivery of services to all patients is delayed in times of crowding, and this may have attributed to the lengthened stay of discharged patients. It is also possible that some patients who initially required inpatient admission improved to the point of discharge while boarded in the PES, thus negating the need of inpatient admission. Other factors to consider are those pertaining to the quality of observation care, particularly the inappropriate selection of patients and failure to reinforce the target time. One of the quality measures for observation care is a high discharge rate within 24 hours of observation [40–42]. Observation care should have clear treatment goal and endpoint. Patients who are likely to need longer than 24 hours of hospital stay should be directly admitted instead of placed under observation. The target time for observation care is unfortunately often not rigorously followed in Taiwan as there is little financial incentive to reach this target. In this study, 24 percent of the discharged patients stayed in the PES for more than 24 hours, and around 9 percent for more than 48 hours. The decision to admit or discharge the patient under observation should be made by the 24 hours mark in most cases. In reality, the decision making is often delayed. As this study did not separate patients whose disposition was decided after the initial evaluation from those who were placed under observation, future studies are needed to understand the extent of this problem.

The “Alameda Model” described by Zeller et al reported that more than 70% of the patients were successfully discharged in less than 24 hours from the PES [10]. In comparison, only 39.5% of the patients were discharged within 24 hours in this study. One point of contention is that whether the 24-hour target for observation is clinically sound or appropriate since some psychiatric emergencies may take longer than 24 hours to resolve. The Comprehensive Psychiatric Emergency Programs in New York State allow PES to have extended observation capacity up to 72 hours [16]. The extra time of observation care could help avoid short-term hospitalization and increase discharge rate. Though it remains unclear if the benefits of extended observation outweigh short-term hospitalization as crowding could be exacerbated by extended LOS. Additional studies are needed to answer these questions.

Although data for PES crowding are scant, compared to previous studies of psychiatric patients in general ED [4,37,38], the rate of prolonged LOS (26.5%) was exceedingly high in this study. This may be a reflection of the health insurance system in Taiwan. As stated before, the lack of financial penalties of the current health insurance policies may be a likely influence. The quality standards set by the National Health Insurance indicate that the proportion of ED patients with LOS longer than 48 hours should be less than 5%. In contrast, the proportion of this study sample with LOS of longer than 48 hours was 7.5%, and the National Health Insurance standard was clearly not met. One of the objectives of the current study was moving towards a solution to this problem which has also plagued many other hospitals in Taiwan. In addition to improving access to inpatient beds, facilitating timely discharge of patients who are not admitted should not be overlooked when making future healthcare policies.

Visits that took place on work days made up roughly 5/7 of the study sample which corresponds to the workweek in Taiwan. Day shift and evening shift visits were overrepresented in the study sample which is also consistent with our clinical experience at the study hospital. Patients arrive during night shifts often stay longer because of decreased staffing at night. The average LOS could have been even longer if there were more night shift visits during the study period.

Associations between arrival by ambulance and use of restraints, and prolonged LOS had been found among psychiatric patients in previous studies conducted in general ED [4,37,38]. This is consistent with the associations observed in this study. These characteristics could be viewed as indicators of acuity and severity, and the result does not lend support to the hypothesis that ED crowding is caused by unnecessary or inappropriate visits [3]. Because this subset of patients are usually more agitated and had a higher risk of aggression or self-injury, they are in more urgent need of treatment. That being the case, these patients carry a significantly higher risk and are difficult to manage in any circumstances. The challenges are magnified in emergency settings due to the physical limitations and the lack of consistency in staffing [34].

Arrival in restraints and eventual referral for involuntary hospitalization both suggest an unwillingness to receive psychiatric treatment. Under Taiwan’s mental health laws, patients may either be held in the PES or transferred to acute care units after temporary psychiatric hold is initiated. Unfortunately, most psychiatric hospital protocols dictate that until requests for involuntary hospitalization are made, all patients under the temporary hold remain in the PES. Requests for involuntary hospitalization must be made by the end of the second day of temporary psychiatric hold, and clinicians often hold off making this decision, thus resulting in prolonged LOS.

Use of illicit substances may induce a plethora of psychiatric symptoms and may also exacerbate a preexisting psychiatric disorder. Many times the decision for inpatient admission is delayed because it is possible that these symptoms would subside rapidly after cessation of substance use. However, this may not always be the case, and these symptoms could be protracted or even respond poorly to medication treatment [43].

First time visit to the hospital was found to be associated with LOS longer than 48 hours. Whereas it did not reach statistically significant difference for LOS longer than 24 hours, the result was close to significant difference (p = 0.07). This may be due to the fact that more time is needed for evaluation and obtaining medical and psychiatric history because of unfamiliarity with these patients. This phenomenon may reflect the inherent limitations of new patient assessment [44].

Our study has certain strengths. To the best of our knowledge, this is the most comprehensive study to date investigating factors associated with prolonged LOS in the PES. The inclusion of all PES patients and random selection reduced the risk of selection bias. Furthermore, electronic data were utilized, thus reducing the human error factor sometimes seen in manual chart reviews.

Interpretations of these findings need to take the following limitations into consideration. This was a retrospective study; therefore information bias and residual confounding remain a possibility. Also, this study was performed at a single institution, and as such, has inherent limitations of generalizability. The sample size was relatively small compared to other studies. Data were limited to those collected for clinical care and not all possible confounding factors were accounted for.

## Conclusion

As illustrated by our study, despite the many advantages it provides, a PES still faces the problem of crowding similar to a general ED. Boarding is a well-known factor that causes prolonged LOS in the general ED [35] and is often the focus of proposed solutions to ED crowding [18,26]. However, in this study transfer to an inpatient unit was not significantly associated with prolonged LOS, and discharged patients had an equally high rate of prolonged LOS as admitted patients. Inappropriate selection of patients and failure to reinforce the target time for observation care are some of the possible explanations for this phenomenon. The results of this study have alerted us to the possibility that factors associated with prolonged LOS in the PES may not necessarily be the same as those associated with prolonged LOS among psychiatric patients in the general ED. Further studies are warranted to tell whether these results are applicable to PES in other countries or unique to Taiwan’s healthcare system and mental health legislation.

Shortage of inpatient beds is often a hospital-wide or region-wide problem that cannot be solved within the PES. On the other hand, improving PES processes to facilitate timely discharge may be equally important and can be achieved with changes made within the PES. Likewise, focus on the target time of observation care should be emphasized and encouraged. Patients who are unlikely to be safely discharged within 24 hours should be directly admitted. Observation care should not be used solely as a means of delaying decision making for disposition.

The results of this study also informed us of the characteristics of patients with prolonged LOS in the PES. Use of restraints and history of illicit substance use were more common among these patients. It is our belief that efforts made to address the needs of these patients would improve the quality of care delivered within the PES.

## Supporting information

S1 File 20180608_Raw data.xlsx. (XLSX)

S2 File 20180608_Study protocol (Chinese version).doc. (DOC)

S3 File 20180608_Study protocol (English translation).docx. (DOCX)

S4 File 20180608_Data extraction form (English translation).docx. (DOCX)
