# Supplementary material for: Factors associated with prolonged length of stay in the psychiatric emergency service
Source: PLoS One. 2018 Aug 20;13(8):e0202569. doi: 10.1371/journal.pone.0202569 (PMC6101399; doi:10.1371/journal.pone.0202569)
Supplement: S2 File — (DOC) [file pone.0202569.s002.doc]

衛生福利部桃園療養院

**臨床研究計畫書**

1. 計畫名稱：急診留觀超過48小時之預測因子分析
2. 研究機構：衛生福利部桃園療養院
3. 計畫摘要：

利用病歷及電子資料庫回顧方式，針對本院過去五年急診就診病患的基本資料（年齡、性別、婚姻狀態、居住狀態、教育程度、工作狀態等）以及臨床資料（到院方式、是否為初診、過去住院次數、精神科主診斷、內外科病史、物質濫用史、是否轉至病房住院治療、是否申請強制住院治療等）進行統計分析，試圖找出影響於急診留觀超過24以及48小時的預測因子。

1. 計畫緣由：

近年來各個醫院的急診單位所面臨的重大問題之一為急診壅塞，而造成壅塞的病患中以精神科病患為其大宗^1^。這個問題已經受到許多關注，因為急診壅塞不只會影響醫院的人力及其他資源的運用，甚至也會影響病人的預後，包括增加隔離、約束和鎮靜藥物的使用機率，以及增加不良事件的發生機率^2,3,4,5^。

本院為精神科專科醫院，且設有封閉式急診，來診病患特徵原本就與一般綜合醫院急診因精神科問題就診之病患不同，且本院近年來急診來診病患特徵也與過去逐漸不同，由警消強制送醫的病患所佔的比率逐年上升，本研究為病歷回顧研究，選取於民國100年1月至104年12月至本院急診就診的病患，分析病患於急診留觀超過24以及48小時的預測因子。

目前國內並無精神科醫院急診留觀病患特性的相關統計，且如果能由收集到的資料中找出急診留觀超過24以及48小時的預測因子，可以幫助我們更有效的預測病患的照護需求，同時也是改善急診壅塞的第一步。

1. 研究目的：
   1. 得知精神科醫院急診就診病患的基本及臨床資料分佈。
   2. 分析精神科醫院急診就診病患於急診留觀超過24以及48小時的預測因子。
2. 研究方法：
3. 受試者選擇標準（納入、排除條件）與數目：
   1. 納入條件：於民國100年1月至104年12月至本院急診就診

的病患。

- 1. 排除條件：無。

(三) 受試者數目：急診就診人次約10000人次，以50抽1的方式

進行抽樣，總計約200人次。

1. 研究設計與進行方法：
   1. 進行方式：利用病歷及電子資料庫回顧方式，針對急診就診病患的年齡、性別、婚姻狀態、居住狀態、教育程度、工作狀態、到院方式、是否為初診、過去住院次數、精神科主診斷、內外科病史、物質濫用史、是否轉至病房住院治療及於急診留觀時間等進行統計分析。
   2. Primary outcome：病患於急診留觀是否超過24小時; secondary outcome：病患於急診留觀是否超過48小時。
   3. 是否有對照組:否。
   4. 盲化方式：無。
   5. 隨機分派：無。
2. 研究之評估與統計方法：
   1. 使用SPSS 20.0 統計軟體。
   2. 以描述性統計分析急診就診病患的基本及臨床資料。
   3. 針對各個不同的基本及臨床資料，以Pearson's chi-squared test（類別變項）或Student's t-test（連續變項）比較於急診留觀超過或未超過24以及48小時的病患間的差異。
   4. 以logistic regression分析急診留觀超過24以及48小時的預測因子。
3. 後續或追蹤計劃：

未來可參考研究成果，繼續進行相關研究。

1. 研究人力及相關設備需求：
2. 研究人力：

| 類別 | 姓名 | 現任職務 | 本計畫內擔任之工作 |
| --- | --- | --- | --- |
| 主持人 | 許竣棋 | 主治醫師 | 負責研究設計、監督計畫執行及論文撰寫。 |
| 專任研究助理 | 陳立萍 | 一般精神科 研究助理 | 協助病歷回顧、資料收集、將結果輸入電腦。 |
|  |  |  |  |
|  |  |  |  |
|  |  |  |  |
|  |  |  |  |
|  |  |  |  |
|  |  |  |  |

1. 相關設備需求：

| 項 目 名 稱 | | 用 途 及 說 明 | 金 額 | 備 註 |
| --- | --- | --- | --- | --- |
| 無 | |  |  |  |
|  | |  |  |  |
|  | |  |  |  |
|  | |  |  |  |
|  | |  |  |  |
|  | |  |  |  |
|  | |  |  |  |
|  | |  |  |  |
|  | |  |  |  |
| 共 計 |  | |  |  |

1. 研究對象權益之保障、同意之方式及內容：

1.所有病歷回溯之個案，皆為例行醫療常規治療之案例。

2.所有病歷回溯之個案資料收集，包括例行醫療及追蹤，皆為在計畫提出日前已完成之病歷。

1. 可能傷害之預防、評估與因應：

1. 計畫主持人及相關研究人員對個案資料負完全之保密責任。

2. 於資料分析時，受試者的身分皆以代碼表示，且保證於任何時期絕

不揭露受試者個人隱私與基本資料。

1. 預期成果及主要效益：

1. 經由研究調查，瞭解本院過去5年急診病患的臨床特徵及變化趨勢。

2. 根據研究結果，瞭解本院急診病患留觀超過24以及48小時的預測因子，並提出建議，作為日後擬定促進醫療品質計畫之參考。

3. 從中學習臨床研究之設計與執行，累積臨床研究之經驗。

1. 研發成果之歸屬及運用：

1.研究成果歸屬於研究計畫主持人及桃園療養院

2.研究成果運用包括：

(1)醫學期刊投稿

(2)臨床應用

1. 研究期間與進度：

|  | 第  一  月 | 第  二  月 | 第  三  月 | 第  四  月 | 第  五  月 | 第  六  月 | 第  七  月 | 第  八  月 | 第  九  月 | 第  十  月 | 第  十一  月 | 第  十二月 | 備 註 |
| --- | --- | --- | --- | --- | --- | --- | --- | --- | --- | --- | --- | --- | --- |
| 準備工作 | ● |  |  |  |  |  |  |  |  |  |  |  |  |
| 資料收集 | ● | ● | ● | ● | ● | ● |  |  |  |  |  |  |  |
| 資料分析 |  |  |  |  | ● | ● | ● | ● | ● | ● | ● | ● |  |
| 撰寫報告 |  |  |  |  |  |  |  |  |  |  | ● | ● |  |
| 預定進度 | 10% |  |  |  |  |  | 50% |  |  | 80% |  | 100% |  |

1. 研究經費來源及其需求：
2. 研究經費來源：院內研究經費
3. 研究經費需求：

| 本年度經費需求：本計畫本年度所需各項經費，請依照「衛生福利部經費補助使用範圍及標準表」詳實編列，各經費項目請務必按照該標準表內所訂之名稱與次序填寫，說明欄內應詳細說明估算方法及用途，並依據會計科目分類。 | | |
| --- | --- | --- |
| 項 目 | 金 額 | 說 明 |
| 人事費 | 79033元 | 專任研究助理（合聘）2.5個月薪資(65000元)、勞退金(4770元)、勞保金(5660元)、健保費(3602.5元) |
| 文具紙張 | 15967元 | 紙張、文具、資料夾、印表機碳粉匣（墨水匣）、感光筒等費用 |
| 其他 | 5000元 | 倫理審查費用 |
|  |  |  |
|  |  |  |
|  |  |  |
|  |  |  |
|  |  |  |
|  |  |  |
|  |  |  |
|  |  |  |
|  |  |  |
|  |  |  |
|  |  |  |

1. 相關文獻：
2. Bender D, Pande N, Ludwig M. A Literature Review: Psychiatric Boarding. Washington, D.C.: U.S. Department of Health and Human Services; 2008
3. Nicks BA, Manthey DM. The impact of psychiatric patient boarding in emergency departments. Emerg Med Int 2012;2012:360308
4. Singer AJ, Thode HC, Jr., Viccellio P, et al. The association between length of emergency department boarding and mortality. Acad Emerg Med 2011;18:1324-9.
5. El-Mallakh RS, Whiteley A, Wozniak T, et al. Waiting room crowding and agitation in a dedicated psychiatric emergency service. Ann Clin Psychiatry. 2012 May;24(2):140-2.
6. Rhodes SM, Patanwala AE, Cremer JK, et al. Predictors of Prolonged Length of Stay and Adverse Events among Older Adults with Behavioral Health-Related Emergency Department Visits: A Systematic Medical Record Review. J Emerg Med. 2016 Jan;50(1):143-52.

衛生福利部桃園療養院

**臨床研究主持人暨協同人個人資料**

106.01.01訂定

■研究主持人 □協同人員

|  | 姓名: 許竣棋 性別: 男 出生年月日: 69年 1月 6日 | | | | | | |
| --- | --- | --- | --- | --- | --- | --- | --- |
| 學 歷 | 學校名稱 | | 在校年月日 | | 主修學科 | | 學位 |
|  | 陽明大學 | | 87年9月至94年6月 | | 醫學系 | | 學士 |
|  |  |  |  |  |  |  |  |
|  |  |  |  |  |  |  |  |
| 經 歷 | 機關名稱 | | 工作單位 | | 職位 | | 在職年月日 |
|  | 桃園療養院 | | 一般精神科 | | 主治醫師 | | 103年4月至今 |
|  |  |  |  |  |  |  |  |
| 近五年曾參與之專題研究計劃 | 研究名稱 | | 擔任工作 | | 起迄年月 | | 補助機關 |
|  |  |  |  |  |  |  |  |
|  |  |  |  |  |  |  |  |
| 附送近五年著作與研究報告 | 名 稱 | 刊物名稱 | | 卷 期 | 頁 次 | 出版時間 | 出 版 地 點 |
|  |  |  |  |  |  |  |  |
|  |  |  |  |  |  |  |  |
|  |  |  |  |  |  |  |  |
|  |  |  |  |  |  |  |  |

衛生福利部桃園療養院

**臨床研究計畫執行單位同意證明**

**106.01.01訂定**

| 計畫名稱 | 急診留觀超過48小時之預測因子分析 |
| --- | --- |
| 計畫執行地點 | 衛生福利部桃園療養院 |
| 資料收集方法 | 本研究利用病歷及電子資料庫回顧方式，針對急診就診病患的年齡、性別、婚姻狀態、居住狀態、教育程度、工作狀態、到院方式、是否為初診、過去住院次數、精神科主診斷、內外科病史、物質濫用史、是否轉至病房住院治療及於急診留觀時間等進行統計分析。 |
| **計畫主持人申請單位**：  本單位同意此案向衛生福利部桃園療養院倫理委員會提出申請。  此致  倫理委員會  計畫主持人：許竣棋 單位：一般精神科 職稱：主治醫師  簽名： 日期：  單位主管：詹宏裕 單位：一般精神科  簽名： 日期： | |
| **執行/收案單位**（同申請單位，本欄位免填）：  本單位同意此案於本單位執行/收案。  此致  倫理委員會  本院科主任或授權代表同意簽署(需由機構首長或授權代表同意簽署)  單位： 職稱：  簽名： 日期： | |
