# Supplementary material for: Factors associated with prolonged length of stay in the psychiatric emergency service
Source: PLoS One. 2018 Aug 20;13(8):e0202569. doi: 10.1371/journal.pone.0202569 (PMC6101399; doi:10.1371/journal.pone.0202569)
Supplement: S3 File — (DOCX) [file pone.0202569.s003.docx]

TAOYUAN PSYCHIATRIC CENTER

**Clinical Research Proposal**

1. Study name:

Predictive factors of emergency room boarding in a psychiatric hospital

1. Study summary

The study sample will be randomly selected from emergency department visits of the past five years. Demographic (e.g. age, gender, marital status, living arrangement, education level and employment status) and clinical (e.g. mode of arrival, history of previous visits, number of previous inpatient admissions, primary psychiatric diagnosis, physical comorbidity, history of substance misuse, transfer to inpatient unit and request for involuntary hospitalization) characteristics will be obtained by both extracting electronic records from the health information system and manually reviewing charts. The aim of the study is finding factors associated with emergency department length of stay over 24 and 48 hours.

1. Introduction

Crowding in the emergency department has become a widespread problem in recent years, and psychiatric patients tend to stay longer in the emergency department than other patients. This has received much attention because emergency department crowding not only exhausts hospital resources but also negatively affects patient outcome, including increased use of seclusion, restraint and sedation, and a higher probability of adverse events.

As this hospital is a specialized psychiatric hospital with a locked emergency department, patient profiles should be different from emergency departments of general hospitals. Also emergency department patient profiles at this hospital have gradually been changing over the years as the proportion of patients arriving in ambulance, with or without police escort has been increasing.

Currently there are limited data on patient profiles of emergency departments in psychiatric hospitals in Taiwan. The proposed study is a retrospective study analyzing emergency department visits from January 2011 through December 2015 to determine factors associated with length of stay over 24 and 48 hours. The search for factors associated with emergency department length of stay over 24 and 48 hours would give us a better understanding of the needs of emergency patients, and it would also be the first step towards solving emergency department crowding.

1. Purpose
   - 1. To clarify the demographic and clinical characteristics of patients visiting psychiatric emergency services
     2. To find factors associated with length of stay over 24 and 48 hours in psychiatric emergency services
2. Methods
3. Selection criteria and estimated number of participants
   1. Inclusion criteria

Visited the emergency department of this hospital at any point from January 2011 through December 2015

- 1. Exclusion criteria

None

- 1. Estimated number of participants

The emergency department annual census is around 10000 visits. About 200 visits will be randomly selected.

1. Study design
   - - 1. Patient characteristics including age, gender, marital status, living arrangement, education level, employment status, mode of arrival, history of previous visits, number of previous inpatient admissions, primary psychiatric diagnosis, physical comorbidity, history of substance misuse, transfer to inpatient unit and length of stay will be obtained by both extracting electronic records from the health information system and manually reviewing charts.
       2. Primary outcome: emergency department length of stay over 24 hours; secondary outcome: emergency department length of stay over 48 hours.
2. Statistical analysis
3. Data will be analyzed using SPSS version 20.0.
4. Descriptive statistics will used to describe the demographic and clinical characteristics of patients.
5. Pearson’s chi-squared test (for categorical variables) and Student’s t-test (for continuous variables) will be used for univariate comparisons.
6. Logistic regression analysis will be used to find factors associated with emergency department length of stay over 24 and 48 hours
7. Protection of participants’ rights
8. All the data the study will use were initially collected as part of routine clinical care.
9. All the medical records the study will use were completed prior to this study proposal.
10. Strict confidentiality will be maintained by the principal investigator and related personnels.
11. All patient data will be fully anonymized before any analysis begins.
