# Supplementary material for: Factors associated with prolonged length of stay in the psychiatric emergency service
Source: PLoS One. 2018 Aug 20;13(8):e0202569. doi: 10.1371/journal.pone.0202569 (PMC6101399; doi:10.1371/journal.pone.0202569)
Supplement: S4 File — (DOCX) [file pone.0202569.s004.docx]

**Factors associated with prolonged emergency department length of stay**

Case No.: Filled out by:

| 1. Gender: □0.Male □1.Female 2. Age: □□□ years 3. Marital status： □0.Never been married □1.Married □2.Divorced □3.Widowed □4.Unclear 4. Living arrangement: □0.Lived alone □1.Lived with family or friends □2.Lived in an institution □3.Unclear 5. Education level:□0.Elementary school □1.Middle school □2.High school □3.College □4.Graduate school 6. Employment? □0.No □1.Yes 7. Primary psychiatric diagnosis (ICD-9-CM code) 8. Psychiatric comorbidity? □0.No □1.Yes 9. Physical comorbidity? □0.No □1.Yes 10. Year of the visit: □□□□ 11. Date of the visit: □□ 12. Arrived on a weekend or holiday? □0.No □1.Yes 13. Time of the visit (hh:mm): □□:□□ 14. Arrived during night shift (24:00-08:00)? □0.No □1.Yes 15. Mode of arrival: □0.Alone □1.Accompanied by family □2.Accompanied by someone other than family □3.By ambulance without police escort   □4. By ambulance with police escort □5.Transferred from another hospital  □6.Transferred from an institution □7.Other   1. Arrived in restraint? □0.No □1.Yes 2. Use of restraint in the ED? □0.No □1.Yes 3. Triage level: □0.I □1.II □2.III □3.IV □4.V □5.Not recorded 4. Transfer to inpatient unit? □0.No □1.Yes 5. Request for involuntary hospitalization? □0.No □1.Yes 6. Age of onset: □□ years 7. New patient? □0.No □1.Yes   If “yes”, please go to question 22.   1. Age of first hospital visit: □□ years 2. Number of previous inpatient admissions: □□ times 3. History of self-injury? □0.No □1.Yes 4. History of violence? □0.No □1.Yes 5. Family history of psychiatric disorders? □0.No □1.Yes 6. History of alcohol misuse? □0.No □1.Yes 7. History of cigarette smoking? □0.No □1.Yes 8. History of illicit substance use? □0.No □1.Yes 9. History of prescription drug misuse? □0.No □1.Yes 10. Length of stay (hh:mm): □□:□□ 11. Length of stay > 24 hours? □0.No □1.Yes 12. Length of stay > 48 hours? □0.No □1.Yes | □  □□□  □  □  □  □  □□□.□□  □  □  □□□□  □□  □  □□:□□  □  □  □  □  □  □  □  □□  □  □□  □□  □  □  □  □  □  □  □  □□:□□  □  □ |
| --- | --- |
